# Compressive fatigue properties of an acidic calcium phosphate cement—effect of phase composition

**DOI:** 10.1007/s10856-017-5851-5

**Published:** 2017-01-31

**Authors:** Ingrid Ajaxon, Caroline Öhman Mägi, Cecilia Persson

**Affiliations:** Materials in Medicine, Division of Applied Materials Science, Department of Engineering Sciences, Uppsala University, The Ångström Laboratory, Box 534, SE-751 21 Uppsala, Sweden

## Abstract

Calcium phosphate cements (CPCs) are synthetic bone grafting materials that can be used in fracture stabilization and to fill bone voids after, e.g., bone tumour excision. Currently there are several calcium phosphate-based formulations available, but their use is partly limited by a lack of knowledge of their mechanical properties, in particular their resistance to mechanical loading over longer periods of time. Furthermore, depending on, e.g., setting conditions, the end product of acidic CPCs may be mainly brushite or monetite, which have been found to behave differently under quasi-static loading. The objectives of this study were to evaluate the compressive fatigue properties of acidic CPCs, as well as the effect of phase composition on these properties. Hence, brushite cements stored for different lengths of time and with different amounts of monetite were investigated under quasi-static and dynamic compression. Both storage and brushite-to-monetite phase transformation was found to have a pronounced effect both on quasi-static compressive strength and fatigue performance of the cements, whereby a substantial phase transformation gave rise to a lower mechanical resistance. The brushite cements investigated in this study had the potential to survive 5 million cycles at a maximum compressive stress of 13 MPa. Given the limited amount of published data on fatigue properties of CPCs, this study provides an important insight into the compressive fatigue behaviour of such materials.

## Introduction

Bone tissue has a certain ability to heal by itself, however if a fracture has reached a critical size, or if the bone tissue is of poor quality, bone grafting may be needed. The global market for bone grafts and substitutes was estimated to almost 2.4 billion US$ in 2014 [1]. With an aging population and an increasing number of patients suffering from osteoporosis the bone graft substitute market is anticipated to continue to rise [2].

Although autologous tissue is still considered the gold standard, it has some drawbacks including donor-site morbidity and supply limitation, thus the market for synthetic materials is steadily growing. Among the synthetic bone grafting materials, calcium phosphate cements (CPCs) are an interesting alternative since they can be degradable, are osteoconductive and can be directly injected into the fracture site [3, 4]. Currently there are several commercial calcium phosphate based formulations available [5]. Limiting factors for an increased use of CPCs in fracture stabilization and as a bone-void filling material, include a strong tradition to use autografts, restricted knowledge about different synthetic alternatives [6], and, perhaps most importantly, their mechanical properties [5].

A recent review on ceramic-based cements and putties pointed out that not only the average compressive strength of a ceramic cement is of importance when characterizing the mechanical properties, but also the strength distribution and the fatigue properties [5]. Moreover, since some bone cement formulations degrade in physiological solutions, the change in cement properties over time needs to be studied. The subject of long-term degradation in terms of quasi-static compressive strength and porosity has previously been addressed by the authors [7]. However, there is a scarcity of data on the fatigue properties of calcium phosphate-based bone cements.

To the best of the authors’ knowledge there is no study available on the dynamic mechanical properties of acidic calcium phosphate cements, i.e., brushite (dicalcium phosphate dihydrate) or monetite (dicalcium phosphate anhydrous), and only four studies on the dynamic mechanical properties of other self-setting calcium phosphate-based cements alone [8–11]. Zhao et al. studied a hydroxyapatite cement under cyclic four-point bending, at 5 Hz, and showed that the cements failed immediately when stresses ≥6 MPa were applied, whereas they survived for 2 million cycles (run-out limit) when the cements were subjected to stress levels ≤5 MPa (corresponding to ≤half of the average quasi-static three-point flexural strength) [8]. Harmata et al. tested a commercially available biphasic bone cement (PRO-DENSE^®^, 75 wt% calcium sulphate and 25 wt% brushite with *ß*-tricalcium phosphate (*ß*-TCP) granules), under compressive fatigue at stress levels between 5–15 MPa, using a frequency of 5 Hz and a run-out limit of 1 million cycles [9]. They found that the median fatigue life was 23,500 cycles when a maximum stress level of 5 MPa was used, whereas the median fatigue life was 236 cycles at 10 MPa, and 4 cycles at 15 MPa. No data on the quasi-static properties of the biphasic bone cement were reported. Fatigue crack growth in calcium phosphate cements has also been studied, by Jew et al. (commercially available apatite cement, name of supplier was not specified) and Morgan et al. (Norian^®^ SRS^®^, an apatite-based cement) [10, 11]. The crack growth threshold value was found to be between 0.08 and 0.11 MPa√m for the two cements, taken at a crack growth rate of 10^−9^ m/cycle.

There are also a few publications on the fatigue properties of CPCs together with human bone, for instance inside vertebrae [12] and in the tibial plateau [13]. In the study by Wilke et al., Calcibon^®^, (apatite cement), was injected ex vivo into osteoporotic human vertebrae and subjected to cyclic loading using a frequency of 5 Hz and a load amplitude between 100 to 600 N. The experimental set-up allowed for complex loading of the cement-bone specimens, including both flexion and bending. The authors concluded that the strength of osteoporotic vertebral bodies could be maintained during cyclic loading corresponding to daily walking for a period of about 3 months (run-out was set to 100,000 cycles), when the calcium phosphate cement was used as an augmenting material [12]. The quasi-static strength of Calcibon^®^ was not reported. McDonald et al. compared Callos^®^ (apatite cement) as a bone augmentation material to an autologous bone graft for human tibial plateau fractures and found that both materials survived all the way to run-out (210,000 cycles). However, the tibial plateau displacement was significantly lower for calcium phosphate-augmented repairs than for those made with the autograft (in average 1.8 vs. 3.3 mm), and the ultimate quasi-static load was significantly higher (in average 2241 vs. 1717 N for calcium phosphate repairs and autografts, respectively). Thus, they concluded that the higher fatigue strength of the calcium phosphate repairs, would increase the immediate load-bearing capabilities of the tibial plateau compared to if autografts would be used. The fatigue study was performed using a frequency of 4 Hz, at a maximum load representing an in situ load from one to three times body weight.

A synthetic vertebral bone augmentation model, comprising a polyurethane foam cube with a cylindrical hole, has also been used to study the fatigue performance of KyphOs™ (apatite cement) [14]. Lewis et al. found that the cement had a 100% probability of surviving 1 million load cycles (run-out) when a maximum compressive stress of 1.7 MPa was used, whereas when the load was doubled the probability was only 28% (median fatigue life 721,919 cycles). All fatigue tests were conducted using a frequency of 3 Hz. The quasi-static compressive strength of the cement alone was not reported.

The fatigue properties of CPCs inside animal bone have also been evaluated. Gisep et al. studied Norian^®^ SRS^®^ and a calcium sulphate cement in a gap defect in sheep tibia and found that there were no differences between the two materials and that cracks started to form after 800 cycles, in both materials, when loads between 1.5–2 kN were applied [15]. The average quasi-static failure load was reported to be 3.7 kN for both Norian^®^ SRS^®^ and calcium sulphate. The frequency used for the fatigue tests was not specified.

The fatigue studies performed on CPCs alone, or in combination with bony tissue, up until now are very limited. Moreover, there are no fatigue studies available at all on CPCs where the main phase is the result of an acidic reaction. This is unfortunate, since recent studies have indicated two clinical advantages of these types of cements compared to apatite cements: faster resorption rates [3, 16, 17] and osteoinductive effects [18, 49, 50], permitting faster bone regrowth. Furthermore, brushite and monetite have previously been found to display different quasi-static strengths [19, 20], and it is hence likely that also the fatigue properties of the cements will differ.

CPCs may be used for direct injection, but also as a pre-set material in bone scaffolds [18, 19]. However, in brushite cements the phase composition is only metastable, and may evolve over time. It is therefore important to also investigate the influence of storage time on the phase composition and the mechanical properties of the cement.

The purpose of this study was to investigate the fatigue behaviour of acidic CPCs. In order to evaluate the effect of phase composition on the fatigue properties and quasi-static strength of the cements, specimens containing different amounts of monetite were investigated.

## Materials and methods

### Cement preparation

The powder phase of the cement consisted of 60 mol% *ß*-TCP (Sigma-Aldrich, St. Louis, MO, USA) and 40 mol% monocalcium phosphate monohydrate (MCPM; Scharlau, Sentmenat) with the addition of 1 wt% disodium dihydrogen pyrophosphate (SPP; Sigma-Aldrich, St. Louis, MO, USA). SPP was added to the powder mixture to prolong the setting time of the cement [21]. The powder phase was manually mixed with double distilled water at a liquid to powder (L/P) ratio of 0.4 ml/g for 30 s. The cement paste was cast into rubber moulds (6 mm in diameter) and left to set for 24 h at 37 °C and 100% humidity. To achieve specimens with plane parallel end surfaces, the set specimens were polished with SiC paper to a final height of 12 mm (specimen dimensions according to the standard ASTM F 451-08 [22]). The cement specimens were stored in desiccators at room temperature (21 ± 1 °C) until further testing after 24 h–240 days.

### Specimen examination

All fabricated specimens were visually examined for external defects. Internal defects were visualized in 2D by micro computed tomography (SkyScan 1172, Bruker microCT, Kontich, Belgium) and measured with the control software of the scanner. Specimens having external or internal defects with a diameter larger than 1 mm were rejected for fatigue testing in accordance with ASTM F 2118-03 [23]. The rejection ratio, i.e., the ratio between the number of discarded specimens and the total number of specimens, was on average 63%. The rejection ratio prior to fatigue testing has not been reported for CPCs before, but is within the range found in the literature for poly(methyl) methacrylate cements (PMMA, 40–72%) [24–27].

### Phase characterization

The phase composition of the specimens was analysed with X-ray diffraction (XRD), using a D8 Advance (Bruker, AXS GmbH, Karlsruhe, Germany) in a theta-theta setup using Ni-filtered Cu-Kα irradiation. The specimens were ground and homogenized and diffraction data was collected from six powder specimens taken at random from this quantity of fine powder. Diffraction angles 2*θ* of 10–60° were analysed in steps of 0.02 degrees with 0.25 s per step, while rotating the sample at a speed of 80 rpm. Profex (http://profex.doebelin.org) [28] in combination with BGMN (http://www.bgmn.de) [29, 30] was used for Rietveld refinement of the powder XRD data. Crystalline models were taken from PDF# 04-013-3344 [31] for brushite, PDF# 04-009-3755 [32] for monetite, PDF# 04-008-8714 [33] for *β*-TCP and PDF# 04-009-3876 [34] for beta-calcium pyrophosphate (*β*-CPP; a constituent of the as-received *ß*-TCP). No other phases were identified in the diffraction patterns. The repeatability of the quantitative phase composition was taken as 2.77 × standard deviation according to ASTM E177-14 [35, 36].

### Quasi-static compressive strength

The quasi-static strength of the specimens was assessed by compression testing at 1 mm/min using a materials testing machine (AGS-X, Shimadzu, Kyoto, Japan) equipped with a 5 kN load cell. No specimens were excluded from the quasi-static testing due to external or internal defects (see a previous section), as exclusion gave the same average strength (a difference of 0.09–6.0 MPa, i.e. within one standard deviation), but with fewer specimens per group (2–10 specimens rather than 7–24).

### Fatigue testing

Fatigue tests were performed under ambient conditions on specimens that had passed the specimen examination, using a dynamic materials testing system (MTS^®^ Axial 858 Mini Bionix^®^ II, MTS Systems Corp., Eden Prairie, MN, USA) equipped with a 5 kN load cell. A cyclic sinusoidal constant-amplitude compression-compression load was applied to each specimen, starting from a small preload of 0.5 MPa. A frequency of 5 Hz was used and the maximum stress level was set to 13 MPa, based on preliminary testing, and similar to what has previously been used for a calcium sulphate and phosphate-based bone cement [9]. This stress level is also in the range of reported compressive strengths of human trabecular bone (1–23 MPa) [37–39], and higher than what has been suggested for fatigue testing of materials for spinal applications (5 MPa) [40]— one suggested use for ceramic cements in load-bearing applications is vertebroplasty [14, 41]. The specimens were tested until catastrophic failure, i.e., until occurrence of a sudden drop in stress, or a run-out of 5 million cycles, as indicated by the standard for fatigue testing of acrylic bone cements [23].

## Results

### Phase characterization

**Fig. 1:**
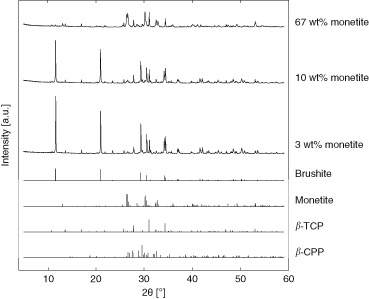
Reference patterns for identified phases and representative XRD patterns of specimens containing different amounts of monetite

**Fig. 2:**
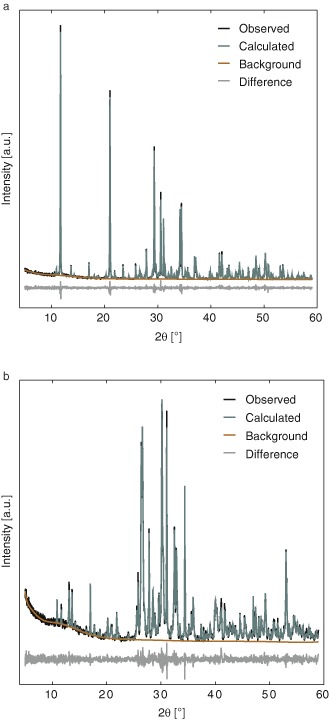
Rietveld refinement of the XRD patterns of **a** specimens containing approximately 3 wt% monetite and **b** specimens containing approximately 67 wt% monetite demonstrates good agreement of the calculated and measured intensities for all four phases. The refinement quality is representative for all datasets included in this study

The Rietveld refinement calculations showed that the cements contained approximately 71 wt% brushite and 3 wt% monetite initially (after 24 h of setting and 1 day of storage), see Figs. 1, 2a. As the amount of monetite increased a concomitant decrease in brushite was seen, and eventually the brushite-to-monetite transformation reached a reversed relationship compared to the starting material (~67 wt% monetite and ~2 wt% brushite), see Figs. 1, 2b. Approximately the same amount of *ß*-TCP (about 20 wt%) and *ß*-CPP (about 7 wt%) could be found in all cement specimens. Repeatability for all phases was better than 1.4 wt%.

### Quasi-static compressive strength

**Fig. 3:**
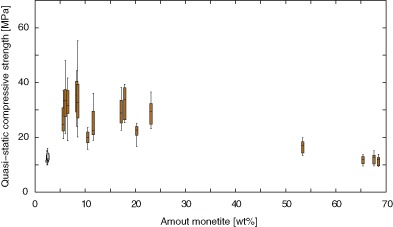
Quasi-static compressive strength as a function of monetite content. Specimens that were tested after 24 h of setting and 1 day of storage are shown as un-filled boxes, all other tested specimens are shown as filled boxes. *n* ≥ 7, per box

The compressive strength for specimens set for 24 h and stored for 1 day, and containing approx. 3 wt% monetite, was on average 14.0 ± 3.5 MPa, see Fig. 3. As the storage time increased and the specimens dried, the quasi-static compressive strength increased twofold, to 29.4 ± 8.0 MPa. These specimens contained up to 23 wt% monetite. For specimens containing higher amounts of monetite (>50 wt%) the quasi-static compressive strength was on average 13.0 ± 2.8 MPa.

### Fatigue

**Fig. 4:**
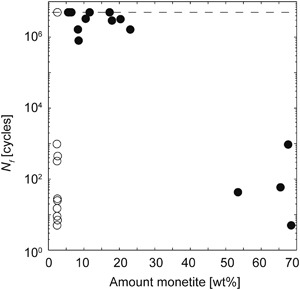
Number of cycles to failure, *N*_*f*_, as a function of monetite content. Specimens that were tested after 24 h of setting and 1 day of storage are shown as un-filled circles, all other tested specimens are shown as filled circles. The dashed line indicates the run-out limit (5 million cycles)

A large scatter in the fatigue results was found, as a function of storage time and monetite content, see Fig. 4. Right after fabrication (24 h of setting and 1 day of storage, specimens containing approximately 3 wt% monetite) most specimens survived between a few cycles up to 1000 cycles, however one specimen survived 5 million cycles (un-filled circles in Fig. 4). Longer storage times allowed for the specimens to dry and led to an increase in the number of cycles to failure, *N*_*f*_, (from approximately 800,000 to run-out) up to a monetite content of approximately 23 wt%. As the brushite-to-monetite conversion progressed and reached levels of 50 wt% monetite, the specimens only survived a few up to 1000 cycles.

## Discussion

In this study, the effect of phase composition of acidic CPCs on the quasi-static compressive strength and compressive fatigue resistance was investigated.

Storing the specimens led to changes in phase composition of the cements, with a transformation of brushite into monetite with time (Fig. 1). It has previously been shown that higher temperatures promote the transformation from brushite to monetite [20, 42]. Moreover, a dry atmosphere is likely to also affect the decomposition. Storing the specimens in desiccators at a stable temperature gave an increase in monetite content over the time of storage (up to 240 days).

The quasi-static compressive strength was affected by a dual effect of both drying and brushite-to-monetite phase transformation (Fig. 3). The strength of CPCs has previously been shown to, in general, be lower for wet specimens compared to dry specimens [43–46]. Specimens containing approximately 3 wt% monetite (specimens set for 24 h and stored for 1 day) were likely still moist when tested in compression, thus resulting in lower strengths. As the cement specimens dry the strength of the cement is expected to be higher, as was demonstrated for specimens containing lower amounts of monetite (up to 23 wt%). However, for specimens having monetite as the main phase the strength was similar to that of the moist specimens (see Fig. 3). Indeed, decomposition of brushite into monetite has been found to increase the porosity of the cement and thus have an adverse effect on the mechanical properties [19, 20, 47].

Fatigue testing of CPCs is prone to result in a large variation in data, due to the inherent porosity of the cements as well as surface flaws. However, both storage and brushite-to-monetite phase transformation had a clear effect on the fatigue results found in this study. The rather low quasi-static strength after 24 h of setting and 1 day of storage resulted in specimens that failed after few loading cycles (with one exception, un-filled circles in Fig. 4), what the authors believe to be an effect of testing specimens that were still moist from fabrication. As already mentioned, as the cements dried, the quasi-static compressive strength of the specimens increased (Fig. 3) and so did the fatigue life (Fig. 4). The specimens that underwent the highest conversion from brushite to monetite were however associated with lower quasi-static strengths (Fig. 3), and resulted in earlier failure of the specimens under dynamic loading (Fig. 4). The fatigue life of the moist cements (those that were tested 24 h after fabrication and 1 day of storage) and of those containing mainly monetite was in the same range as what has previously been found for a calcium sulphate and phosphate-based cement [9] (from a few cycles to a few hundred, using a compressive stress of 10–15 MPa). However, for the cements that were dry, and before the phase transformation from brushite to monetite had progressed too far, the fatigue life presented herein is higher (800,000—5 million cycles for specimens containing up to 23 wt% monetite). Moreover, the run-out limit used in the present study (5 million cycles) is five times longer compared to what Harmata et al. used [9], indicating the potential use of acidic cements in applications where compressive loading over longer periods of time is expected, e.g., certain types of constrained vertebral compression fractures. Indeed, CPCs have successfully been demonstrated to stabilize certain osteoporotic vertebral fractures and found to be a good alternative to PMMA cements both ex vivo [48] and in vivo [41]. It should be noted however, that the current testing was performed in air, and chemical degradation in vivo is likely to have a strong effect on the mechanical properties of the cements, as further discussed below.

The present study made evident that for pre-set cement scaffolds it is important to control the phase composition of the cements, since it can be detrimental to the mechanical performance under quasi-static compression, as well as under compressive fatigue.

One limitation of the study is that the number of tested specimens in fatigue was low and only one maximum stress level was evaluated. On the other hand, results from quasi-static compression and phase composition were based on a larger number of replicates. The authors believe that the clear correlations seen between phase composition, quasi-static strength and fatigue, and the scarcity of fatigue data in the literature of pure CPCs still lend importance to the study.

Self-setting CPCs can either be used as pre-set scaffolds or injected directly into the fracture site. For both applications, the CPC will be in direct contact with physiological fluids when implanted into the body. It has previously been shown that the compressive strength of brushite cements decreased when the cements were soaked either in water, PBS or a serum solution over longer periods of time [7], thus the environment surrounding the material may also have an influence on the compressive fatigue life. Indeed, the fatigue performance of a hydroxyapatite cement has been evaluated at room temperature in physiological fluid [8] and a biphasic cement, where the main phase consisted of calcium sulphate, was tested in dynamic loading under constant hydration [9]. However, none of these studies presented any results obtained under ambient conditions. Moreover, up until now there are no publications at all on the fatigue properties of pure acidic CPCs alone, even though the in vivo loading scenario is dynamic. Therefore, future studies should evaluate fatigue of CPCs under ambient conditions as well as under conditions simulating an in vivo environment, e.g., keeping the cements wet throughout the study and performing the fatigue tests in a physiological solution kept at 37 °C, to be able to investigate the effect of environmental surroundings on the fatigue performance of the cements.

The presence of flaws in the material, both in the form of surface defects and pores, is known to affect the fatigue performance. In this study, specimens having large internal and surface defects were excluded from the fatigue tests, as suggested in the standard for fatigue testing of acrylic bone cements [23]. This exclusion was done in order to decrease the scatter in the experiment, thus simplifying the analysis. Although not evaluated in this specific study, similar cement compositions have shown a porosity of approximately 39% for specimens containing 3 wt% monetite and approximately 56% for specimens containing 55 wt% monetite [42]. The above-mentioned publications on fatigue properties of calcium phosphate-based cements [8–11] did not evaluate the porosity of the cement specimens. In future studies the porosity of each specimen should be evaluated in order to assess any correlation between the porosity and pore size distribution, and the fatigue properties.

## Conclusions

In this study, it was found that pre-set acidic cements have the potential to survive 5 million cycles under compressive-compressive fatigue at a maximum stress of 13 MPa. However, the fatigue properties were affected by both storage of the cements and their phase composition. Moist cements had a lower fatigue resistance compared to cements that were dry, in accordance with quasi-static compressive strength results. Moreover, while cements with brushite as the main phase and monetite amounts of up to approx. 23 wt% did not have a detrimental effect on the fatigue life, specimens having monetite as the main phase gave a clear decrease in fatigue properties. The findings presented herein are of importance for the increased use of acidic CPCs in fracture stabilization and as bone-void filling materials, as they remedy the current lack of data on fatigue performance.
